# Supplementary material for: Dissociation of two-dimensional excitons in monolayer WSe2
Source: Nat Commun. 2018 Apr 24;9:1633. doi: 10.1038/s41467-018-03864-y (PMC5915447; doi:10.1038/s41467-018-03864-y)
Supplement: Supplementary file 1 — Supplementary Information [file 41467_2018_3864_MOESM1_ESM.pdf]

Supplementary Information:
Dissociation of two-dimensional excitons in monolayer WSe_2

Mathieu Massicotte[1], Fabien Vialla[1], Peter Schmidt[1], Mark B. Lundeberg[1], Simone Latini[2], Sten Haastrup[2], Mark Danovich[3], Diana Davydovskaya[1], Kenji Watanabe[4], Takashi Taniguchi[4], Vladimir I. Fal'ko[3], Kristian S. Thygesen[2], Thomas G. Pedersen[5], Frank H.L. Koppens[1]

[1]ICFO – Institut de Ciències Fotòniques, The Barcelona Institute of Science and Technology, Castelldefels (Barcelona) 08860, Spain

[2] CAMD, Department of Physics, Technical University of Denmark, 2800 Kgs. Lyngby, Denmark

[3]National Graphene Institute, University of Manchester, Booth St E, Manchester M13 9PL, UK

[4]DepartmentNational Institute for Materials Science, 1-1 Namiki, Tsukuba 305-0044, Japan

[5]Department of Physics and Nanotechnology, Aalborg University, DK-9220 Aalborg East, Denmark and Center for Nanostructured Graphene (CNG), DK-9220 Aalborg Øst, Denmark

Supplementary Figures

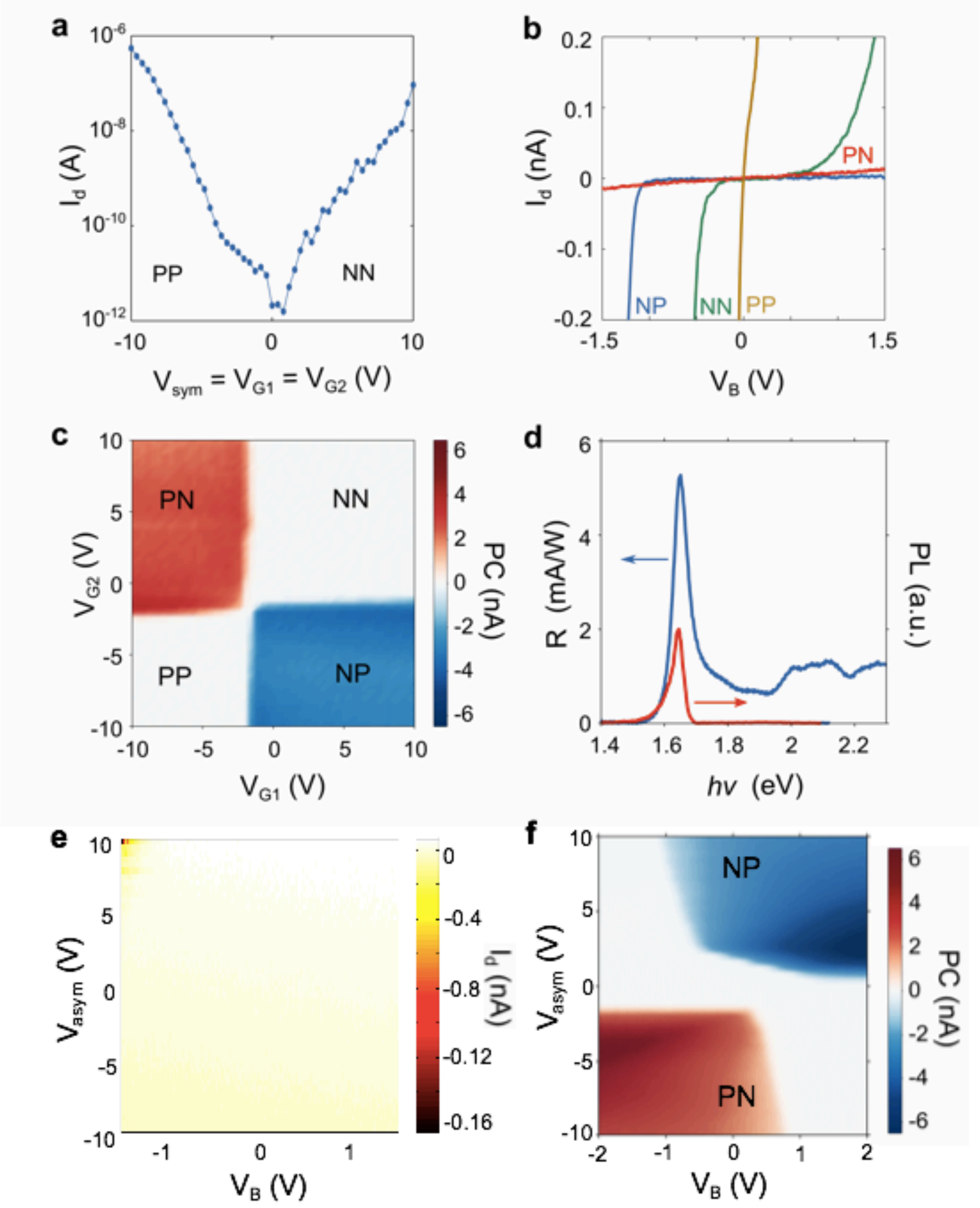

Supplementary Figure 1. Characterization of device 1. **a**) Variation of the dark current I_d as a function of V_{sym} = V_{G1} = V_{G2} at V_B = -1.5 V. **b**) Dark IV curves measured under different gate voltage configurations: n-p (solid blue line; V_{G1} = 10 V, V_{G2} = -10 V), n-n (solid green line; V_{G1} = 10 V, V_{G2} = 10 V), p-p (solid yellow line; V_{G1} = -10 V, V_{G2} = -10 V), p-n (solid red line; V_{G1} = -10 V, V_{G2} = 10 V). **c**) PC measured at the junction as a function of gate voltages V_{G1} and V_{G2} at V_B = 0 V, laser power P = 0.5 μW and photon energy $h\nu$ = 1.65 eV. Each region of the four-fold pattern is identify with the corresponding doping configuration. **d**) Solid blue line: Responsivity (R = PC/P) measured as a function phonon energy $h\nu$ at V_B = 0 V, V_{asym} = 10 V and P = 0.5 μW. Solid red line: Photoluminescence spectrum measured using a CW laser at $h\nu$ = 1.76 eV, V_B = 0 V, V_{asym} = 0 V and P = 200 μW. **e**) Dark current I_d and **f)** PC vs V_{asym} (= V_{G1} = -V_{G2}) and source-drain bias V_B. PC was measured using the same laser parameters as **c**.

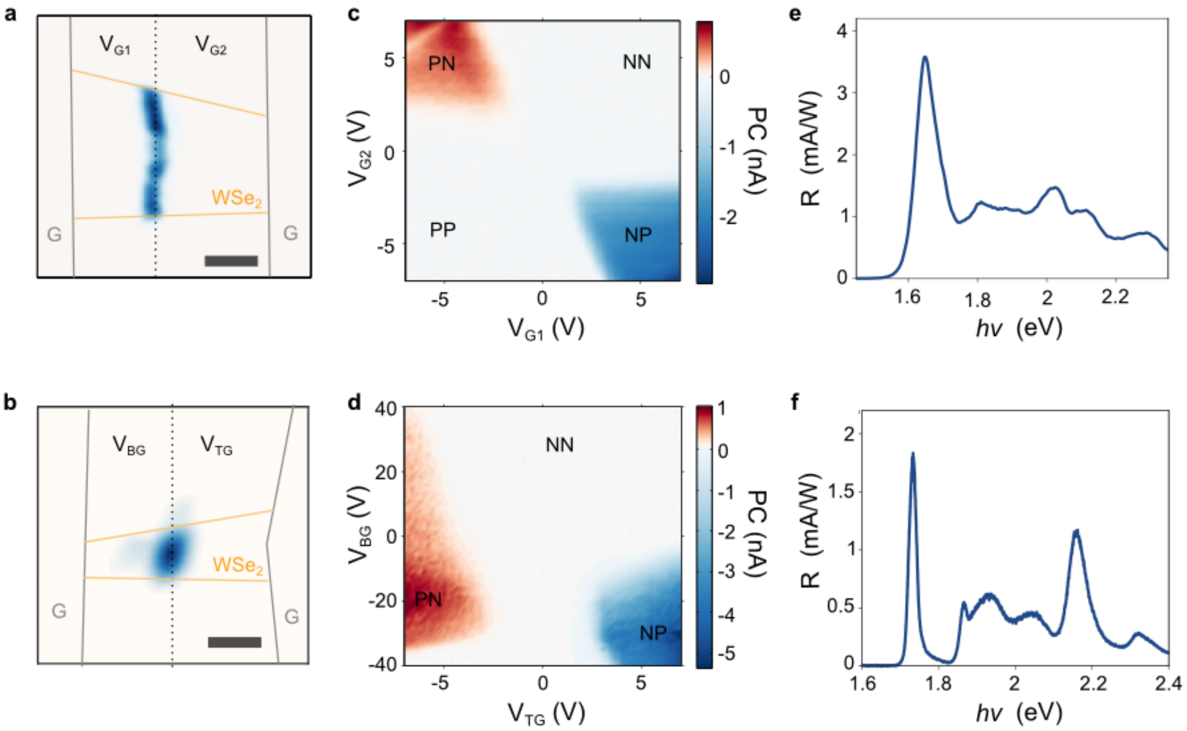

Supplementary Figure 2. Characterization of Device 2 at room temperature (**a**, **c**, **e**) and Device 3 at T = 30 K (**b**, **d**, **f**). All PC measurements were performed at P ~ 1 μW. **a,b**) PC maps of (**a**) Device 2 (V_{asym} = V_{G1} = -V_{G2} = 6 V, $h\nu$ = 1.65 eV) and (**b**) Device 3 (V_{TG} = 7 V, V_{BG} = -30 V, $h\nu$ = 1.72 eV) . The solid grey and orange lines correspond to the edge of the graphite and WSe_2 flakes, respectively. The dotted lines represent the edge of the top gates. The scale bars are 4 μm. **c,d**) PC measured at the junction versus (**c**) gate voltages V_{G1} and V_{G2} of Device 2 at $h\nu$ = 1.65 eV and (**d**) top (V_{TG}) and bottom (V_{BG}) gate voltages of Device 3 at $h\nu$ = 1.73 eV. Both measurements were performed at V_B = 0 V and each region of the four-fold patterns is identify with the corresponding doping configuration. **e,f**) Responsivity (R = PC/P) measured as a function phonon energy $h\nu$ at V_B = 0 V on (**e**) Device 2 (V_{asym} = 7 V) and (**f**) Device 3 (V_{TG} = 7 V, V_{BG} = -30 V).

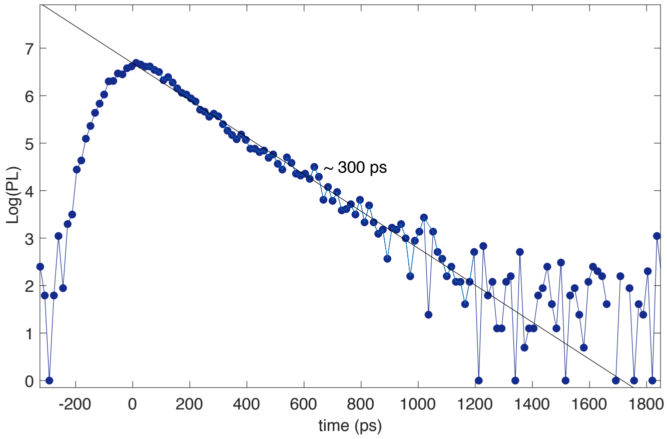

Supplementary Figure 3. Time-resolved photoluminescence of an hBN-encapsulated monolayer WSe_2 at room temperature. The black line is a linear fit yielding a lifetime of ~ 300 ps.

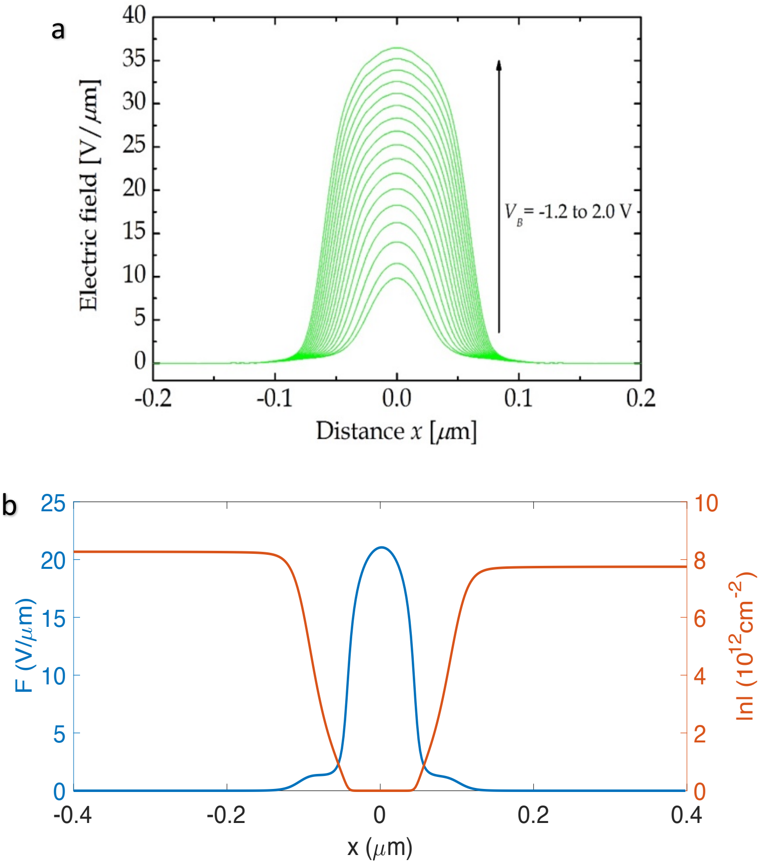

Supplementary Figure 4. a) Distribution of the horizontal electric field vs. V_B for V_{asym} = 10 V. **b**) Spatial distribution of the in-plane electric field F (left axis, blue curve) and charge carrier density n (right axis, red curve) inside the WSe_2 layer calculated for V_{asym} = -10 V and V_B = 0 V.

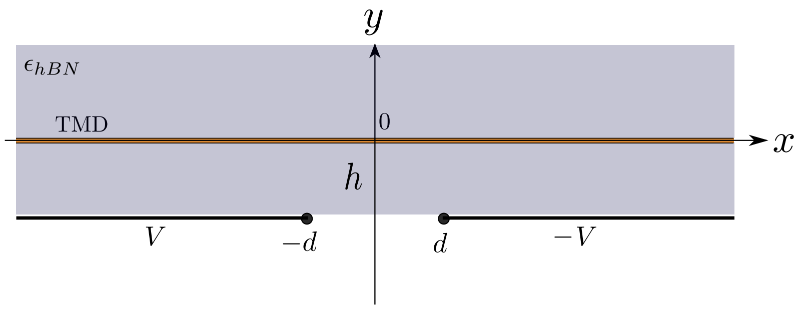

Supplementary Figure 5. Sketch of the system used for the analytical modelling. The TMD layer is encapsulated within two slabs of hBN with a dielectric constant ϵ, and is placed at a distance h from a split gate with opposite voltages $\pm V$ and a separation of $2d$.

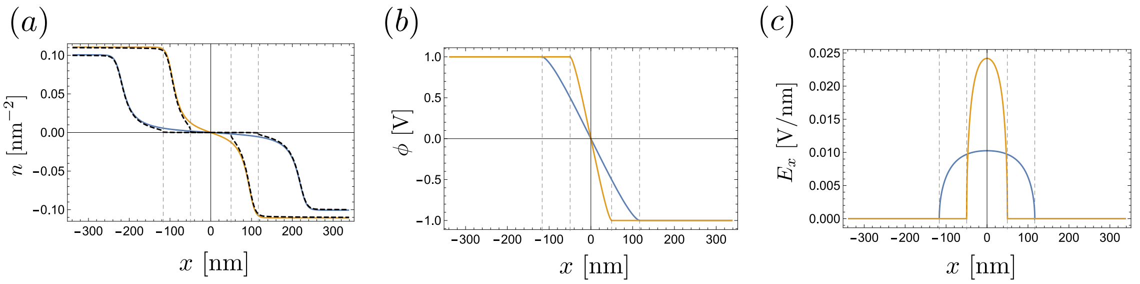

Supplementary Figure 6. Distributions of (**a**) the charge density obtained from the purely electrostatic problem (solid) and including the TMD band structure (dashed), (**b**) the electrostatic potential ϕ, and (**c**) the electric field E_x in the monolayer TMD plane as a function of position x, for two devices (blue and orange). The dashed grey lines mark the boundaries of the incompressible strip. The parameters used are $V = 10\,V, E_g = 2\,eV, \epsilon = 4,$ and the device specific parameters are (blue) $2d = 450\,nm,\ h = 22\,nm,$ and (orange) $2d = 200\,nm,\ h = 20\,nm.$

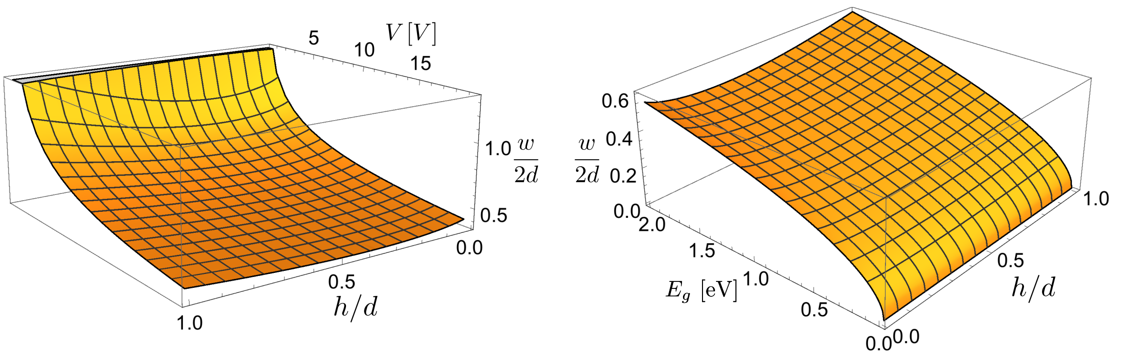

Supplementary Figure 7. (Left) The incompressible strip width w in units of the gates separation $2d$, as a function of the gate voltage V and as a function of $\frac{h}{d}$, (Right) as a function of E_g and $\frac{h}{d}$.

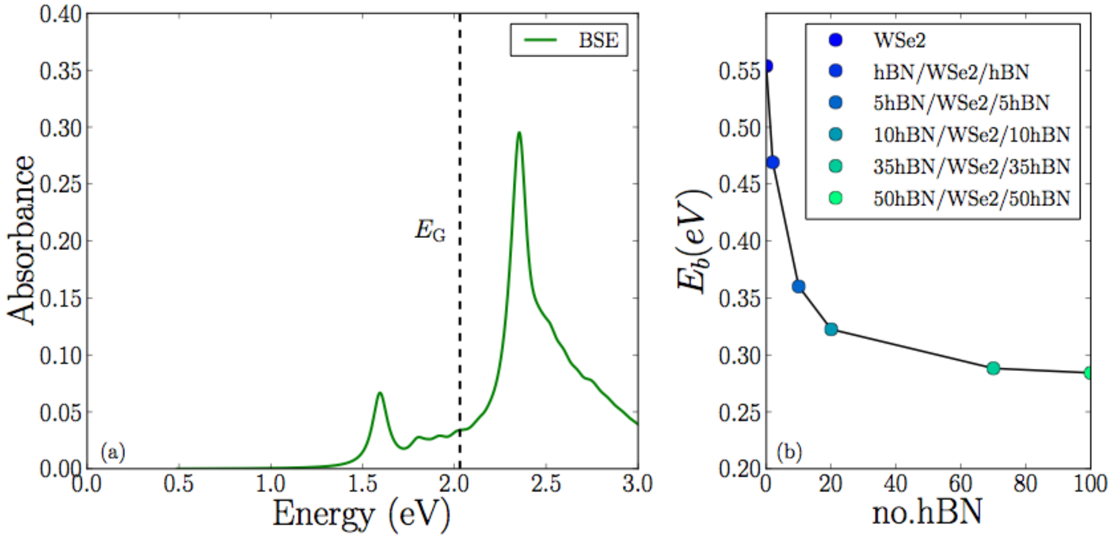

Supplementary Figure 8. **a**) BSE absorption spectrum for a freestanding WSe_2 monolayer, which yield an exciton binding energy of 0.44 eV **b**) Exciton binding energy as a function of the number of the encapsulating hBN layers calculated with the QEH–Mott-Wannier model. The exciton binding energy for the encapsulated case converges to 0.29 eV.

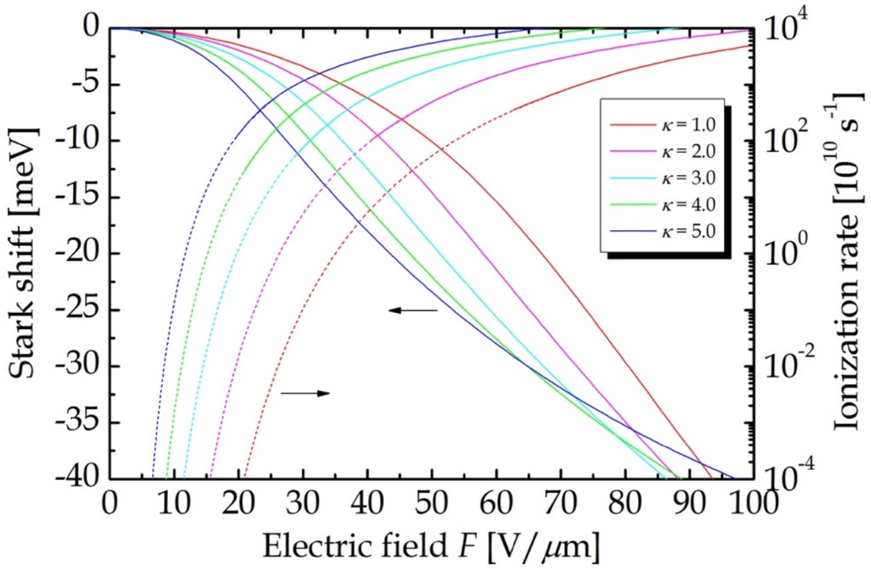

Supplementary Figure 9. Stark shifts and ionization rate for different dielectric encapsulations. Solid lines are complex scaling calculations while dashed are fits to the analytical low-field form.

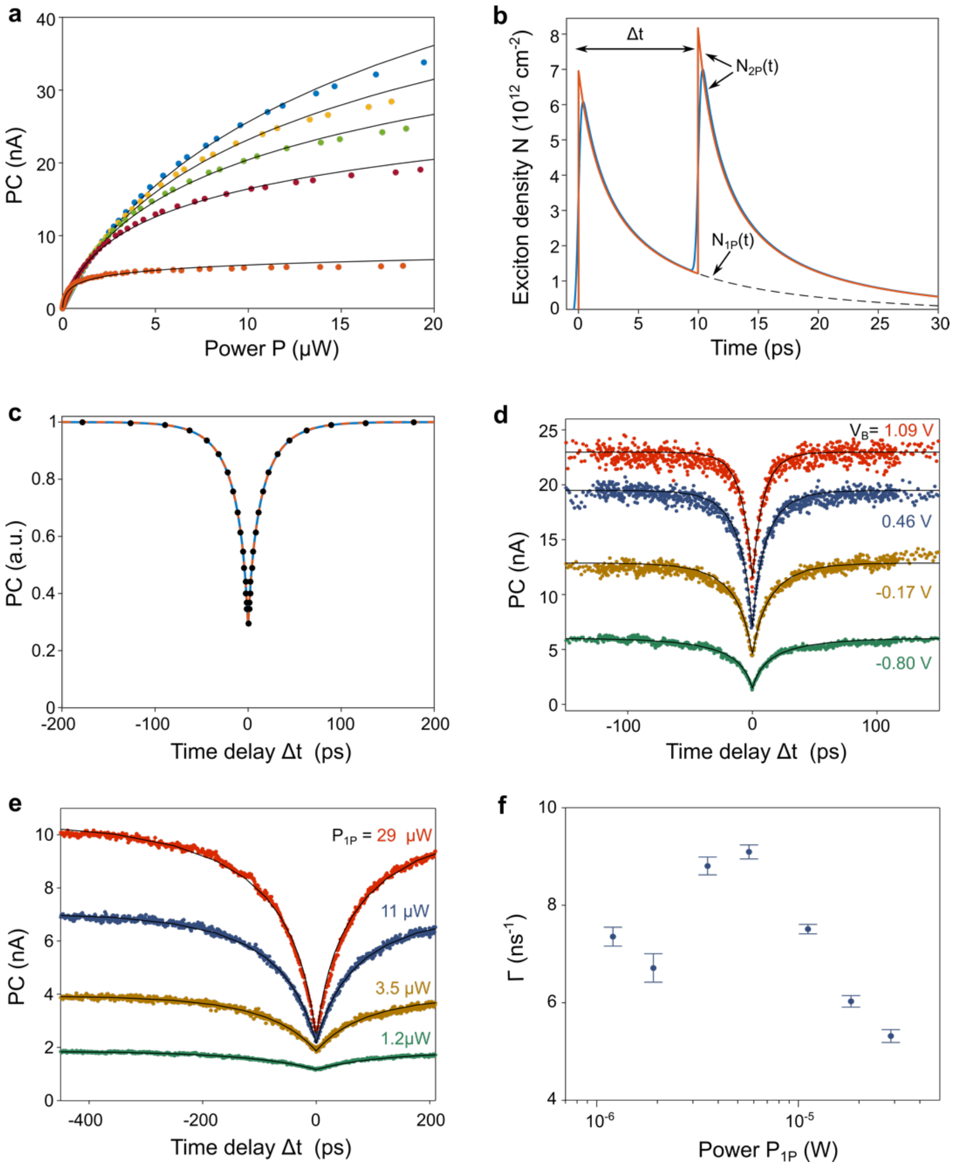

Supplementary Figure 10. **a**) Photocurrent versus laser power P measured at various gate voltages V_{asym} and V_{B} = 0 V. The solid black lines are fit to the data using Supplementary Equation 13. **b**) Temporal dependence of the calculated exciton density $N_{2\mathrm{P}}$ generated by 200 fs-long pulses (solid blue line) and Dirac delta-function pulses (solid red line). The black dotted line represents the single-pulse exciton density $N_{1\mathrm{P}}$. Calculations are carried out using N_0= 7x10^{12} cm^{-2}, Δt = 10 ps, τ = 25 ps and γ = 0.05 cm^{2}s^{-1}. **c**) Calculated PC as a function of the time delay Δt between pulses. The orange dotted line is computed using Dirac delta-function pulses (Supplementary Equations 15 and 16), the blue line is calculated using the analytic expression (Supplementary Equation 17) and the black dots are computed numerically using 200 fs-long pulses. **d**) TRPC measurements at various bias voltages V_{B}, V_{asym} = 10 V and single-pulse, time-averaged power $P_{1\mathrm{P}}$ = 100 μW. **e**) TRPC measurements at different single-pulse power $P_{1\mathrm{P}}$ measured on Device 2 at V_{asym} = 6 V and V_{B} = 0 V. The solid black lines in (**d**) and (**e**) are fit to the data using the model presented in Supplementary Note 5. **f**) Photoresponse rate $\Gamma = 1/\tau$ extracted from the fit shown in (**e**) as a function of single-pulse power $P_{1\mathrm{P}}$. Error bars correspond to the standard deviation obtained from these fits.

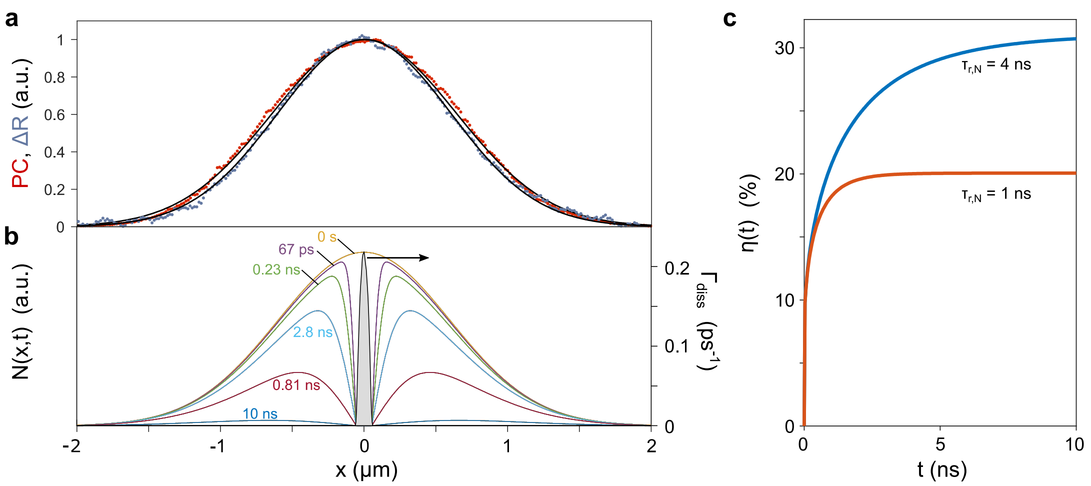

Supplementary Figure 11. **a**) Normalized photocurrent *PC(x)* (red data points) and reflection $\Delta R(x)$ (blue data points) profiles measured by scanning a focused laser beam ($h\nu$ = 1.65 eV, P = 4 μW) on Device 1 with V_B = 0 V and V_{asym} = 10 V. The black lines are Gaussian fits to the data, with standard deviations σ_{PC} = 0.64 μm and $\sigma_{\Delta R}$ = 0.60 μm. **b**) Exciton density $N(x,t)$ calculated using Supplementary Equation 27 for different times between t = 0 and 10 ns, assuming $\tau_{r,N}$ = 4 ns. The junction is located at x = 0 and calculations are performed using the in-plane electric field simulated for V_B = 2 V and V_{asym} = 10 V. The dissociation rate $\Gamma_{diss}(x,F) = 1/\tau_{diss}(x,F)$ is calculated using the model presented in Supplementary Note 4 and represented by the solid black line with grey shading (right axis). **c**) Time evolution of η, the ratio between the number of dissociated excitons and the total number of excitons, calculated for $\tau_{r,N}$ = 1 and 4 ns (solid red and blue lines, respectively).

Supplementary Tables

κ_{hBN}	E_B (meV)	a ($\mu m^{1/2} s^{-1} V^{-1/2}$)	F_0 ($V \mu m^{-1}$)
1	501	6.7×10^{16}	488
2	331	4.84×10^{16}	362
3	261	2.51×10^{16}	261
4	190	1.62×10^{16}	196
4.5	170	1.82×10^{16}	179
5	153	8.46×10^{15}	144

Supplementary Table 1. Exciton binding energy (E_B) and fitting parameters (a and F_0) of the analytical ionization rate obtained for different values of averaged dielectric constant in hBN (κ_{hBN}).

Supplementary Note 1: Device characterization

Measurements shown in the main text mainly come from one device (Device 1, shown in Fig. 1c of the main text). Two other similar devices, Devices 2 and 3, were measured. The photocurrent spectra of Device 3 measured at low temperature (T = 30 K) are shown in Fig. 2a of the main text. In this section we show additional measurements on Device 1 that demonstrate its proper electrical functioning, and we present a basic description and characterization of Devices 2 and 3.

Supplementary Figures 1a,b shows the electrical behavior of Device 1 in the dark at room temperature. The dark current I_d increases as the device is electrostatically doped with electrons ($V_{sym} = V_{G1} = V_{G2} > 0$) and holes ($V_{sym} < 0$), demonstrating ambipolar operation (Supplementary Figure 1a). From this curve, we can estimate the 2-probe field-effect mobility μ_{FE} using the relation

$$\mu_{FE} = \frac{L}{W}\frac{dI_d}{dV_{sym}}\frac{1}{CV_B}, \quad (1)$$

where L and W are the channel length and width, respectively, C is the gate capacitance per unit area ($C = 2\times10^{-7}$ F cm^{-2}) and V_B is the bias voltage used for the measurement. We find that μ_{FE} reaches 3 cm^{2}V^{-1}s^{-1} at V_{sym} = -10 V, in excellent agreement with the mobility extracted from the photoresponse rate measurements. This value also agrees with the field-effect mobilities reported in the literature (μ_{FE} ~ 0.1 to 100 cm^{2}V^{-1}s^{-1})[1,2].

Supplementary Figure 1b shows that the rectifying behavior of the device can be increased by applying gate voltages of opposite polarity, thereby forming a p-n junction. The dependence of the photocurrent (PC) on the gate voltages displays a clear four-fold pattern corresponding to the four possible doping configurations (Supplementary Figure 1c). The fact that photocurrent is measured only in the p-n and n-p configuration indicates that the device functions as a photodiode. The photoluminescence (PL) spectrum measured on the device confirms the monolayer nature of the WSe_2 flake (Supplementary Figure 1d). The PL peak also matches well the A exciton peak observed in the PC

spectrum. Finally, Supplementary Figures 1e,f show the IV characteristics as a function of V_{asym} with and without illumination, respectively.

Devices 2 and 3 are made out of the same hBN/1L-WSe_2/hBN stack, with bottom and top hBN flakes that are 22 and 57 nm thick, respectively. Device 2 is equipped with two top gates separated by ~ 1 µm, while Device 3 only has one top gate covering half of the WSe_2 channel. In this case, we use the heavily doped silicon substrate as a global backgate to control the doping in the other half of the channel. We measured Device 2 at room temperature and Device 3 at T = 30 K. Supplementary Figures 2a,b show the geometry and PC maps of both devices. Like Device 1, the photocurrent displays a characteristic four-fold pattern as gates voltages are varied (Supplementary Figures 2c,d). Finally, the photocurrent spectra (Supplementary Figures 2e,f) of both devices present similar features, but since Device 3 was measured at low temperature, its spectral features are narrower and the entire spectrum is blue shifted compared to the PC spectra of Devices 1 and 2. We note that the difference between the maximum responsivity of these devices is likely due to sample-to-sample variations.

Finally, we measured the exciton lifetime on a sample similar to those used in our study (hBN-encapsulated WSe_2) through time-resolved PL measurements. We extracted an exciton lifetime of ~ 300 ps (Supplementary Figure 3), in reasonable agreement with the value extracted from Fig. 4a of the main text (~ 1 ns).

Supplementary Note 2: Calculation of the electric field distribution

Finite-element calculations

Electric field distributions are calculated using the "electrostatic" and "semiconductor" packages of the finite-element solver COMSOL. In the absence of source-drain bias, the field can be computed from Poisson's equation for a charge slab. For this we assume a temperature of 300 K, a band gap of 1.8 eV, effective masses $m_{\mathrm{e}} = 0.44m_0$ and $m_{\mathrm{h}} = 0.48m_0$, and undoped material. In the presence of a bias, a finite semiconductor thickness is required. We use a

thickness of 5 nm and raise the temperature slightly to 350 K to achieve convergence. By comparison to the unbiased reference these approximations are found to reduce the maximum field by about 10%, for which we then correct all calculations made with source-drain bias. The field distributions for a range of V_B values and V_{asym} = 10 V are shown in Supplementary Figure 4a.

From these calculations, we also obtain the charge density profile $n(x)$ across the p-n junction (right axis of Supplementary Figure 4b). We compared this charge distribution to the in-plane field in the same condition (left axis of Supplementary Figure 4b) and observe that the field is highest in the charge neutral (undoped) zone. Hence, the exciton ionization rate (determined by the ratio E_B/F, see Supplementary Note 4) is always higher in the undoped region, where the exciton binding energy is unaffected by Coulomb screening. This means that the exciton binding energy relevant to our analysis is the one corresponding to undoped WSe_2.

Analytical calculations

The electrostatics of the system shown in Supplementary Figure 5 is modelled by considering the internal energy of the electrons in the TMD layer to be a perturbation to the purely electrostatics problem[3]. This is justified in the limit $\frac{E_g}{eV} \ll 1$, where E_g is the band gap of the TMD monolayer, and V is the voltage on the split gates. Therefore, we first treat the TMD layer as a metal with a constant zero potential and obtain the resulting charge distribution. The electrostatic potential in the region bounded by the gates and the TMD layer, with the specified boundary conditions, is solved using the theory of complex functions and the Schwarz-Christoffel transformation[4], giving the charge density distribution shown in Supplementary Figure 5a by the blue and orange lines for two devices.

The effect of including the internal energy of the charge carriers in the TMD on the electrostatic solution, is the appearance of an incompressible strip of width w, having a constant charge density equal to zero due to the chemical potential

being inside the gap. Using the purely electrostatic solution we now reduce the problem to the TMD plane, where we require a potential drop of E_g to be formed across the strip, in order to bring the valence band above the Fermi energy in the p-side of the p-n junction, and redistributing the charge in the strip to create a constant zero charge density within the strip.

We solve the corresponding Laplace equation in the region $y < 0$ bounded by the TMD plane, with the solution written as a sum of two potentials, $\varphi = \varphi_1 + \varphi_2$, with the boundary conditions given by[5,6]

$$\varphi_1(x) = \begin{cases} \frac{E_g}{2}, & x < -\frac{w}{2}, \\ -\frac{E_g}{2}, & x > \frac{w}{2}, \end{cases}$$
$$\frac{d\varphi_1(x)}{dy} = 0,\ |x| < \frac{w}{2}, \qquad (2)$$
$$\varphi_2(x) = 0,\ |x| > \frac{w}{2},$$
$$\frac{d\varphi_2(x)}{dy} = \frac{2\pi e}{\varepsilon}\left[x n'(x) + \frac{x^3}{6} n^{(3)}(0)\right],\ |x| < \frac{w}{2}.$$

In the last equation, we use the first two terms in the expansion of the charge density $n(x)$ in the strip around $x = 0$, with the gradients of the charge density taken from the purely electrostatic solution.

The solution for the potentials in the TMD which provide a correction to the purely electrostatic solution, are given by

$$\varphi_1(x) = -Re\left\{\frac{E_g}{e\pi}\arcsin\left(\frac{2x}{w}\right)\right\},$$
$$(3)$$
$$\varphi_2(x) = \frac{\pi e}{\epsilon} x\left(\frac{w^2}{4} - x^2\right)[n'(0) + \frac{n^{(3)}(0)}{12}(\frac{w^2}{8} + x^2)], \qquad |x| < \frac{w}{2}.$$

The incompressible strip width is obtained by setting the electric field $E_x = -\frac{d\phi}{dx}$ to be zero at the edges of the strip, thus eliminating the singularity in the electric field at these points. We get for the strip width

$$w^2 = \frac{16n'(0)}{n^{(3)}(0)}\left[\sqrt{1+\frac{E_{\mathrm{g}}\,\epsilon|n^{(3)}(0)|}{2\pi^2e^2n'(0)^2}}-1\right], \tag{4}$$

with the charge density gradients obtained from the purely electrostatic solution,

$$n'(0) = -\frac{2\epsilon pV}{eh^2}\frac{1}{(1+p)^3\left(1+\frac{1}{p}\right)},$$

$$n^{(3)}(0) = -\frac{16\epsilon\pi^2p^4V}{eh^4}\frac{(p-3)(3p-1)}{(1+p)^{10}}, \tag{5}$$

where p is a geometrical parameter obtained by solving the transcendental equation,

$$\frac{d}{h}\pi = \frac{1-p^2}{2p} + \log\frac{1}{p}. \tag{6}$$

In Supplementary Figure 6 we show the resulting charge, potential, and electric field distributions in the TMD plane for two devices. The small correction to the charge density shown in Supplementary Figure 6a by the dashed black lines, following the inclusion of the internal energy of the TMD charge carriers, justifies the assumption made.

Finally, in Supplementary Figure 7 we show the dependence of the incompressible strip width on the gate voltage, the split gate geometry, and the monolayer TMD band gap.

Supplementary Note 3: Ab-initio calculations of the bandgap and exciton binding energy for encapsulated WSe_2

To support the experimental measurement of the electronic bandgap and the exciton binding energy, we provide ab-initio calculations based on the many-body framework of the G_0W_0 approximation and the Bethe-Salpeter Equation (BSE). The G_0W_0 and BSE methods have been successfully employed to calculate

electronic and excitonic excitations in freestanding TMDs[11–17], however the encapsulation of TMDs in hBN crystals makes the use of these methods less trivial. While the direct application of many-body methods to lattice-mismatched multi-layers heterostructures is computationally unfeasible, one can rely on the negligible interlayer hybridization and calculate the excited state properties of the heterostructure from the knowledge of the excited state behavior of the constituting layers. The main challenge is to account for the effect of interlayer electronic screening on the excited state properties of the heterostructure. We have recently addressed this challenge in a series of papers[18–21] by combining our quantum electrostatic heterostructure (QEH) model with the G_0W_0 approach and the 2D Mott-Wannier exciton model. In short, in the QEH model the dielectric response of the heterostructure is calculated by first encoding the dielectric response of each layer in the isolated condition into a dielectric building block, and subsequently coupling the building blocks in a purely electrostatic fashion. Once the dielectric function of the heterostructure is known, the screened electron-electron and electron-hole interactions, the central quantities in many-body theory, can be directly calculated.

Starting with the electronic bandgap calculations, the main idea is to correct the screened interaction, $\bar{W}_{\mathrm{GG'}}^{\mathrm{vdWH}}(\boldsymbol{q},\omega)$ in a G_0W_0 calculation for the WSe_2 layer in the following manner:

$$\bar{W}_{\mathrm{GG'}}^{\mathrm{vdWH}}(\boldsymbol{q},\omega) = \bar{W}_{\mathrm{GG'}}^{\mathrm{monolayer}}(\boldsymbol{q},\omega) + \Delta W(\boldsymbol{q},\omega)\delta_{\mathrm{G0}}\delta_{\mathrm{G'0}} \qquad (7)$$

where $\Delta W(\boldsymbol{q},\omega)$ is the correction due to the extra screening coming from the neighboring hBN layers. Such a correction is calculated by means of the QEH model as the difference between the WSe_2 electron-electron interaction in the encapsulated and freestanding conditions. With this correction the effect of interlayer screening is included at the computational cost of a standard monolayer G_0W_0 calculation. The monolayer G_0W_0 calculations have been performed using the efficient method proposed in ref. 21 that overcomes the problem of slow convergence of the band structures with respect to the k-point

grid and yields well converged band gaps with 18 × 18 k-points. We use an energy cut-off of 150 eV for the calculation of the screened interaction. The G_0W_0 band energies were extrapolated to the limit of infinite number of plane waves.

Our ab-initio calculations including spin-orbit interactions yield a band gap for freestanding the WSe_2 monolayer of 2.03 eV. When the WSe_2 layer is encapsulated in hBN the band gap is reduced to 1.85 eV. We find that such a value does not decrease further if the number of layers of hBN on each side of WSe_2 is increased above 30. The ab-initio value is in good agreement with the measured value of 1.9 eV. The 0.18 eV reduction of the gap by the hBN encapsulation is the signature of the enhanced dielectric screening.

For the exciton binding energy calculations we combined the QEH model with the Mott-Wannier hydrogenic model (see previous Supplementary Note 4). By directly solving the BSE for a freestanding WSe_2 monolayer we find an exciton binding energy of 0.44 eV as it can be seen from the absorption spectrum in Supplementary Figure 8a. Using the Mott-Wannier model for the freestanding WSe_2 we obtain a binding energy of 0.55 eV, which is 0.11 eV higher than the one calculated with the BSE. The difference in the two approaches reflects the limitations of the Mott-Wannier model which is an approximation of the BSE[18]. In the solution of the BSE we use a cut-off energy of 150 eV for the evaluation of the screened interaction and only use the top valence and bottom conduction band for representing the two-particle Hamiltonian. The starting point of the BSE is LDA but a scissor operator based on the G_0W_0 calculations is applied. Spin-orbit effects are not included. The optical absorption spectrum in Supplementary Figure 8a is calculated with a 60 × 60 k-point-mesh. For the Mott-Wannier model we use an exciton effective mass of 0.23 a.u. as calculated from the LDA band structure.

When the WSe_2 layer is embedded in hBN the QEH–Mott-Wannier model yields a binding energy of 0.29 eV, i.e. a 0.26 eV reduction with respect to the freestanding layer case. This can be seen in Supplementary Figure 8b where the

lowest exciton binding energy is reported as a function of the number of encapsulating hBN layers. Since, as shown in previous works[18,19], the reduction in exciton binding energy is well described by the Mott-Wannier model, we can correct the BSE results for the freestanding WSe_2 layer by 0.26 eV and infer that the exciton binding energy for hBN encapsulated WSe_2 is 0.18 eV. This is again in excellent agreement with the experimental value of 0.17 eV.

Supplementary Note 4: Mott-Wannier exciton model

The two-dimensional Wannier equation is applied to model excitons in WSe_2 monolayers. In the presence of an in-plane electric fields, the Wannier equation reads as

$$\left\{-\frac{\hbar}{2\mu}\nabla^2 - w(r) + eFr\cos\theta + E_\mathrm{g}\right\}\psi_\mathrm{exc}(\vec{r}) = E_\mathrm{exc}\psi_\mathrm{exc}(\vec{r}), \tag{8}$$

where $\mu = m_\mathrm{e}m_\mathrm{h}/(m_\mathrm{e}+m_\mathrm{h})$ is the reduced in-plane exciton mass equal to $\mu = 0.23m_0$ with m_0 the free electron mass[7] and E_g is the band gap. The potential $w(r)$ is the screened Coulomb attraction given by the Keldysh form[8]

$$w(r) = \frac{\pi}{2r_0}\left[\mathrm{H}_0\left(\frac{\kappa r}{r_0}\right) - \mathrm{Y}_0\left(\frac{\kappa r}{r_0}\right)\right]. \tag{9}$$

Here, $r_0 = 46.2$ Å is the screening length[7] and $\kappa = (\kappa_\mathrm{a} + \kappa_\mathrm{b})/2$ is the average of the dielectric constants of the surrounding materials above and beneath the sheet. For hBN we use an average between in-plane and out-of-plane dielectric constants[9] $\kappa_\mathrm{hBN} = (4.95 + 4)/2 \approx 4.5$. To simulate excitons with increased and decreased binding energies, we take $\kappa_\mathrm{hBN} = 4.0$ and 5.0 and obtain E_B = 190 and 153 meV, respectively. We compute the exciton susceptibility in the Wannier approximation via the expression

$$\chi(\omega) = \chi_0 \sum \frac{|\psi_\mathrm{exc}(0)|^2}{E_\mathrm{exc}[E_\mathrm{exc}^2 - (\hbar\omega + i\hbar\gamma)^2]}, \tag{10}$$

where χ_0 is a material dependent constant. Also, the sum is over all exciton states with wave function $\psi_{\text{exc}}(\vec{r})$ and energy E_{exc} relative to the ground state. Finally, $\hbar\gamma = 15$ meV is a phenomenological line shape broadening. Absorption is simulated as $\omega Im\chi(\omega)$. The eigenstates are found by expanding in a Bessel function basis $J_m(\lambda_{mn}r/R)\cos(m\theta)$, where J_m is the m'th Bessel function of the first kind and λ_{mn} is its n'th zero, i.e. $J_m(\lambda_{mn}) = 0$. The parameter R is a large but finite confinement radius introduced to restrict the problem to a finite area. We use $R = 500$ in units of the effective exciton Bohr radius, a basis of 7 different angular momenta ($m = 0, \ldots, 6$), and 400 states ($n = 1, \ldots, 400$) for each value of m.

To calculate exciton ionization rates we use the complex scaling technique. This consists in substituting $\vec{r} \to e^{i\phi}\vec{r}$ in Supplementary Equation 8, which then becomes non-Hermitian and has complex eigenvalues $E = E_0 + \Delta - \frac{i}{2}\Gamma$ with E_0 the unperturbed eigenvalue, Δ the Stark shift, and Γ the ionization rate. We take $\phi = 0.2$ and apply a Sturmian-Laguerre basis with $m = 0, \ldots, 6$ and $n = 1, \ldots, 150$. The ionization rate can be reliably computed for relatively large fields, i.e. $F \geq 15$ Vμm^{-1}. To extrapolate to smaller fields, we fit computed values in the range 18 to 24 Vμm^{-1} to the expected form for a two-dimensional material $\Gamma = aF^{-1/2}\exp(-F_0/F)$ using a and F_0 as fitting parameters[10] (see Supplementary Table 1). Stark shifts and ionization rates for three different dielectric surroundings are shown in Supplementary Figure 9.

Supplementary Note 5: Nonlinear photoresponse model

Power dependence of the photocurrent

Recently, several studies have investigated the nonlinear optical response of TMDs and revealed a complex picture. The physical origin of this nonlinearity has been attributed to different processes, including phase space filling[22], bandgap renormalization[23] and exciton-exciton annihilation[24]. In order to simplify the analysis of the nonlinear photoresponse that we observe, we

consider only one photoexcited specie (excitons) and introduce a quadratic loss term to the kinetic rate equation:

$$\frac{dN}{dt} = G(t) - \frac{N}{\tau} - \gamma N^2 \tag{11}$$

where N is the exciton density, $G(t)$ is the exciton generation rate, τ is the photoresponse time, γ is the time-independent exciton-exciton annihilation (EEA) rate. Under pulsed excitation (e.i., $G(t) = N_0\delta(t)$, where N_0 is the initial exciton density), the solution to Supplementary Equation 11 is :

$$N(t, N_0) = \frac{N_0 \exp\left(\frac{-t}{\tau}\right)}{1 + \gamma\tau N_0\left(1 - \exp\left(\frac{-t}{\tau}\right)\right)} \tag{12}$$

In our experiment, the photocurrent we measure is proportional to the time-integrated excitons density:

$$PC(N_0) \propto \int_0^\infty N(t, N_0)\, dt$$
$$\propto \frac{1}{\gamma}\ln\left(1 + \gamma\tau N_0\left(1 - \exp\left(\frac{-t}{\tau}\right)\right)\right)\Bigg]_0^\infty = \frac{1}{\gamma}\ln(1 + \gamma\tau N_0) \tag{13}$$

As can be seen in Supplementary Figure 10a (and Figs. 3a,b of the main text), this simple expression provides a good model for the sublinear power dependence of the photocurrent. We fit this Supplementary Equation 13 to our data by assuming an initial exciton density is

$$N_0 = \frac{A}{f\pi r_{\mathrm{L}}^2 h\nu} P \tag{14}$$

where $A \sim 5\%$ is the absorption coefficient, P is the time-averaged laser power, $f = 40$ MHz is the laser repetition rate, $r_{\mathrm{L}} = 0.7$ μm is the laser radius and $h\nu =$

1.65 eV is the photon energy. From these fits, we extract the value of $\gamma\tau$ as a function of V_{asym} and V_B shown in Fig. 3c of the main text.

Time-resolved photocurrent (TRPC) measurements

We employ the TRPC technique to determine the absolute values of τ and γ, and thereby disentangle the contribution of the linear and nonlinear processes to the photoreponse of our device. This technique consists in measuring the photocurrent produced by two ultrashort (~ 200 fs) laser pulses with the same fluence as a function of the time delay Δt between the two pulses. In our experiment, one of the two pulses (the pump) is modulated by a mechanical chopper, such that the photocurrent (measured by lock-in) is:

$$PC(\Delta t) \propto \int_0^\infty N_{2P}(t, N_0, \Delta t)\, dt - \int_0^\infty N(t, N_0)\, dt \tag{15}$$

where $N(t, N_0)$ is the exciton density produced by a single pulse with initial exciton density N_0 (Supplementary Equation 14), and $N_{2\mathrm{P}}(t, N_0, \Delta t)$ is the exciton density produced by two pulses delayed by a time Δt. Assuming instantaneous (Dirac) pulses, this function can be written as

$$N_{2\mathrm{P}}(t, N_0, \Delta t) = \begin{cases} N(t, N_0) & \text{for } 0 < t < \Delta t \\ N\big(t - \Delta t, N_0 + N(\Delta t, N_0)\big) & \text{for } t > \Delta t \end{cases} \tag{16}$$

Supplementary Figure 10b shows $N_{2\mathrm{P}}(t)$ and $N(t)$ calculated for N_0= 7x10^{12} cm^{-2}, Δt = 10 ps, τ = 25 ps and γ = 0.05 cm^{2}s^{-1}. We also calculate $N_{2\mathrm{P}}(t)$ produced by pulses with finite duration (200 fs) and find Supplementary Equation 16 to be a good approximation. Using Supplementary Equation 15, we numerical solve $PC(\Delta t)$ for both finite duration pulses and Dirac delta-function pulses (Supplementary Figure 10c) and observe no significant difference between both solutions.

In order to fit this model to our TRPC measurements, we develop an analytical expression that mimics the numerical solution. This expression takes into account the two limits of $PC(\Delta t)$:

$$PC(\Delta t \rightarrow \infty) \propto 2\int_0^\infty N(t, N_0)\, dt - \int_0^\infty N(t, N_0)\, dt = \frac{1}{\gamma}\ln(1 + \gamma\tau N_0)$$

$$PC(\Delta t = 0) \propto \int_0^\infty N(t, 2N_0)\, dt - \int_0^\infty N(t, N_0)\, dt = \frac{1}{\gamma}\ln\left(1 + \frac{\gamma\tau N_0}{1 + \gamma\tau N_0}\right)$$

Interpolating between these two limits gives us the following analytical expression:

$$PC(\Delta t) \propto \ln\left[1 + \frac{\gamma\tau N_0}{1 + \gamma\tau N_0}\left(1 + \gamma\tau N_0\left(1 - \exp\left(\frac{-\Delta t}{\tau}\right)\right)\right)\right] \qquad (17)$$

We compare this expression to the numerical solutions of $PC(\Delta t)$ for the same set of parameters (Supplementary Figure 10c) and observe very good agreement. Supplementary Equation 17 also indicates that $PC(\Delta t)$ is governed by the exponential term $\exp\left(\frac{-\Delta t}{\tau}\right)$, which justifies the single exponential decay model often used to interpret TRPC measurements.

We employ this analytical expression (Supplementary Equation 17) to fit our TRPC measurements and extract τ and other relevant parameters. The fitting parameters are then used to calculate the numerical solution of Supplementary Equation 15 for pulses with finite duration. These numerical solutions are represented by the solid black lines in Supplementary Figures 10d,e (and Figs. 3d,e of the main text) and appear to match very well with the TRPC measurements.

Finally, and importantly, we note that this model provides a way to determine the intrinsic value of τ independently of the laser power P employed for the TRPC measurements. Indeed, the model accounts for changes in the initial exciton density N_0 generated by a laser power P via Supplementary Equation 14. This

represents a significant advantage over conventional models using a single exponential decay[25] which typically yield power-dependent τ. Analysis of the TRPC measurements as a function of laser power using our model is shown in Supplementary Figures 10e,f. No obvious trend between τ and P is observed when P is varied over more than one order of magnitude. We find however that the extracted value of τ varies by ~40%, which might be a result of our fitting procedure.

Supplementary Note 6: Linear photoresponse model

Here we present the details of the photoresponse model that we introduced in the main text to explain the measured field-dependent photoresponse rate Γ and internal quantum efficiency (IQE). Our model describes the linear response of our device at low power excitation ($P \lesssim 1$ µW). In this regime, exciton-exciton annihilation and other nonlinear processes are negligeable. This assumption is justified since the values of Γ (extracted using the nonlinear model presented in Supplementary Note 5) are disentangled from nonlinear effects and associated with linear processes. The linear response assumption also applies to the IQE because it was measured at low power (P = 0.5 µW).

As explained in the main text, our model considers that excitons generated in (or diffusing to) the p-n junction at a rate G can either recombine at a field-independent rate $\Gamma_{\mathrm{r},N} = 1/\tau_{\mathrm{r},N}$ or dissociate by tunnel ionization at a rate $\Gamma_{\mathrm{diss}} = 1/\tau_{\mathrm{diss}}$. Prediction and calculations for Γ_{diss} as a function of in-plane electric field are presented in Supplementary Note 4. Upon dissociation, free carriers drift out of the junction at a rate $\Gamma_{\mathrm{drift}} = 1/\tau_{\mathrm{drift}} = 2\mu F_{\mathrm{avg}}/L$, where μ is the free carrier mobility and L is the junction length. F_{avg}, the average electric field across the junction, is calculated from the field simulations presented in Supplementary Note 2. The drift process competes with the field-independent recombination of free carriers which occurs at a field-independent rate $\Gamma_{\mathrm{r},n} = 1/\tau_{\mathrm{r},n}$. Hence, the exciton density N and free carrier density n in the junction can be described by the following coupled rate equations:

$$\frac{dN}{dt} = G - \frac{N}{\tau_{\text{diss}}} - \frac{N}{\tau_{\text{r},N}} = G - \frac{N}{\tau_N} \tag{18}$$

$$\frac{dn}{dt} = \frac{N}{\tau_{\text{diss}}} - \frac{n}{\tau_{\text{drift}}} - \frac{n}{\tau_{\text{r},n}} = \frac{N}{\tau_{\text{diss}}} - \frac{n}{\tau_n} \tag{19}$$

Where $\Gamma_N = \frac{1}{\tau_N} = \frac{1}{\tau_{\text{diss}}} + \frac{1}{\tau_{\text{r},N}}$ and $\Gamma_n = \frac{1}{\tau_n} = \frac{1}{\tau_{\text{drift}}} + \frac{1}{\tau_{\text{r},n}}$ represent the total exciton and free carrier decay rates, respectively. The overall photoresponse rate Γ of our device is determined by the slowest of these two rates and can therefore be approximately expressed as

$$\Gamma = \frac{1}{\tau} \approx \frac{1}{\tau_N + \tau_n} \tag{20}$$

As we show in the main text, Supplementary Equation 20 captures well the field dependence of the measured photoresponse rate.

We now consider the efficiency of the photocurrent generation process predicted by our model. Under steady state conditions (i.e. CW illumination), the photocurrent produced is proportional to the rate at which free carriers drift out of the p-n junction, such that

$$PC \propto \frac{dn_{\text{extract}}}{dt} = \frac{n}{\tau_{\text{drift}}} \tag{21}$$

Where n_{extract} is the density of free carriers extracted out of the junction. Setting the time derivatives of Supplementary Equations 18 and 19 to zero, we find the following relation between the rate at which excitons are created and the rate at which free carriers are extracted:

$$\frac{dn_{\text{extract}}}{dt} = \frac{\tau_n}{\tau_{\text{drift}}} \frac{\tau_N}{\tau_{\text{diss}}} G = \eta_{\text{extract}} G \tag{22}$$

where η_{extract}, the extraction efficiency, can be rewritten as

$$\eta_{\mathrm{extract}} = \frac{\tau_{\mathrm{r},n}}{\tau_{\mathrm{r},n} + \tau_{\mathrm{drift}}} \frac{\tau_{\mathrm{r},N}}{\tau_{\mathrm{r},N} + \tau_{\mathrm{diss}}} = \eta_{\mathrm{drift}}\, \eta_{\mathrm{diss}} \tag{23}$$

From this expression, it is clear that the extraction efficiency characterizes how efficiently excitons generated in (or diffusing to) the p-n junction dissociate into free carriers (η_{diss}), and how efficiently theses free carriers drift out of the junction (η_{drift}).

Supplementary Note 7: Collection efficiency

In this section, we address the diffusion and collection excitons generated outside the junction. Indeed, in our experiment, a significant fraction of the total exciton population is generated away from the junction because the laser spot size we use is larger than the junction length. In order to contribute to the photocurrent, these excitons first need to diffuse to the junction. Those who reach the junction then undergo the extraction process described in Supplementary Note 6. The total IQE is therefore given by

$$\mathrm{IQE} = \eta_{\mathrm{coll}}\, \eta_{\mathrm{extract}} \tag{24}$$

where η_{extract} is the extraction efficiency (see Supplementary Note 6), and η_{coll}, the collection efficiency, is define as the ratio between the number of excitons reaching the p-n junction and the total number of excitons generated (or photons absorbed) in the WSe_2. η_{coll} can be evaluated using the following equation:

$$\eta_{\mathrm{coll}} = \frac{\iint G(x,y)\, CP(x,y)\, dxdy}{\iint G(x,y)\, dxdy} \tag{25}$$

where $G(x,y)$ is the exciton generation rate and $CP(x,y)$, the collection probability, corresponds to the probability that an exciton generated in a certain

region of the device has to reach the junction. In what follows, we evaluate η_{coll} in our experiment using two different approaches.

Experimental estimation of η_{coll}

The first method consists in estimating the collection probability CP from the measured laser spot and photocurrent profile. Considering that the width of the junction (in the y-direction) is much wider than the laser spot size, the photocurrent profile *PC(x)* obtained by scanning the laser position across the junction (in the x-direction) corresponds to the convolution of $G(x)$ and (x) :

$$PC(x) \propto \int G(x - x')\, CP(x')dx' \quad (26)$$

To simplify our analysis, we assume that both $G(x)$ and $CP(x)$ can be described by Gaussian profiles characterized by σ_G and σ_{CP}, respectively. Then, according to Supplementary Equation 26, the photocurrent also has a Gaussian profile with $\sigma_{PC} = \sqrt{\sigma_G^2 + \sigma_{CP}^2}$.

Since the exciton generation rate is proportional to the intensity of the laser spot, we determine σ_G by measuring the reflection R of the laser as it is scans across the sharp junction made of two gates separated by 200 nm. Fitting the normalized ΔR with a Gaussian yields $\sigma_G = 0.60 \pm 0.01$ µm, which translates into a FWHM of 1.4 µm (Supplementary Figure 11a). Repeating this procedure with the measured $PC(x)$ gives $\sigma_{PC} = 0.64 \pm 0.01$ µm, which implies that $\sigma_{CP} = \sqrt{\sigma_G^2 + \sigma_{PC}^2} = 0.19 \pm 0.014$ µm. Using these values, we evaluate the collection efficiency with Supplementary Equation 25 and find $\eta_{\text{coll}} = 30 \pm 3\%$, which agrees very well with our IQE measurements and predictions for η_{extract} (see the inset Fig. 4a of the main text).

Numerical estimation of η_{coll}

We can also evaluate η_{coll} by including exciton diffusion to the linear photoresponse model described in Supplementary Note 6. To model the

dynamic of the exciton density $N(x,t)$ across the junction, we numerically solve the following partial differential equation:

$$\frac{\partial N(x,t)}{\partial t} = D_N \frac{\partial^2 N(x,t)}{\partial x^2} + G(x,t) - \frac{N(x,t)}{\tau_{\mathrm{diss}}(x,F)} - \frac{N(x,t)}{\tau_{\mathrm{r},N}} \quad (27)$$

where D_N is the exciton diffusion constant, $G(x,t)$ is the exciton generation rate, $\Gamma_{\mathrm{diss}}(x,F) = 1/\tau_{\mathrm{diss}}(x,F)$ is the field-dependent exciton dissociation rate and $\Gamma_{\mathrm{r},N} = 1/\tau_{\mathrm{r},N}$ is the field-independent exciton recombination rate. We assume that excitons are generated by a Gaussian laser pulse, such that $G(x,t) \propto \delta(t) f(x, \sigma_G = 0.60\ \mu\mathrm{m})$, where f represents a Gaussian function.

To evaluate η_{coll}, we calculate the total number of excitons that dissociate at the junction, $N_{\mathrm{diss}}(t)$, and compute

$$\eta = \frac{N_{\mathrm{diss}}(t \to \infty)}{\int G(x,y)\, dx} = \eta_{\mathrm{coll}} \eta_{\mathrm{diss}} \quad (28)$$

η_{coll} can be estimated by considering a case where the in-plane field *F(x)* is high, such that $\eta_{\mathrm{diss}} = \frac{\tau_{\mathrm{r},N}}{\tau_{\mathrm{r},N} + \tau_{\mathrm{diss}}(F)} \sim 1$ and $\eta \sim \eta_{\mathrm{coll}}$. For this purpose, we use *F(x)* simulated for V_B = 2 V and V_{asym} = 10 V (see Supplementary Note 2) and calculate $\tau_{\mathrm{diss}}(x,F)$ using the model presented in Supplementary Note 4 (Supplementary Figure 11b).

Finally, D_N and $\tau_{\mathrm{r},N}$ are set according to the values we extracted from our analysis of photoresponse time presented in the main text. Specifically, the diffusion coefficient is evaluated using $D_N = \frac{\mu_N k_B T}{e_0}$, where μ_N is the exciton mobility, k_B is Boltzmann's constant, T = 300 K is the ambient temperature and e_0 is the elementary charge. For our simulations, we assume that μ_N is equal to the free carrier mobility (μ = 4 $cm^2V^{-1}s^{-1}$) determined experimentally. Since our measurements give a rather imprecise value of $\tau_{\mathrm{r},N}$ ~ 1 ns, we evaluate η_{coll} for

$\tau_{\mathrm{r},N} = 1$ and 4 ns, corresponding to diffusion lengths $L_{\mathrm{D}} = \sqrt{D_N\,\tau_{\mathrm{r},N}} = 0.1$ and 0.2 µm, respectively.

The time evolution of $N(x,t)$ for $\tau_{\mathrm{r},N} = 4$ ns calculated by solving Supplementary Equation 27 is shown in Supplementary Figure 11b. Supplementary Figure 11c shows the increase of η as a function of time for both $\tau_{\mathrm{r},N} = 1$ and 4 ns. From this last figure, we conclude that η_{coll} ~ 20 – 30 %, for diffusion lengths of 0.1 and 0.2 µm, respectively. These values match well with the value of η_{coll} obtained with the experimental approach and the one deduced from our IQE measurements (see main text).

Supplementary References

1. Cui, X. *et al.* Multi-terminal transport measurements of MoS_2 using a van der Waals heterostructure device platform. *Nat. Nanotechnol.* **10,** 534–540 (2015).
2. Wang, Q. H., Kalantar-Zadeh, K., Kis, A., Coleman, J. N. & Strano, M. S. Electronics and optoelectronics of two-dimensional transition metal dichalcogenides. *Nat. Nanotechnol.* **7,** 699–712 (2012).
3. Khaetskii, A. V., Fal'ko, V. I. & Bauer, G. E. W. Electrostatics of inter-Landau-level diodes. *Phys. Rev. B* **50,** 4571–4576 (1994).
4. Weber, E. *Electromagnetic Fields: Theory and Applications*. (1950).
5. Chklovskii, D. B., Shklovskii, B. I. & Glazman, L. I. Electrostatics of edge channels. *Phys. Rev. B* **46,** 4026–4034 (1992).
6. Chklovskii, D. B., Matveev, K. A. & Shklovskii, B. I. Ballistic conductance of interacting electrons in the quantum Hall regime. *Phys. Rev. B* **47,** 12605–12617 (1993).
7. Olsen, T., Latini, S., Rasmussen, F. & Thygesen, K. S. Simple Screened Hydrogen Model of Excitons in Two-Dimensional Materials. *Phys. Rev. Lett.* **116,** 1–5 (2016).
8. Keldysh, L. V. Coulomb interaction in thin semiconductor and semimetal films. *Soviet Physics JETP* **29,** 658–660 (1979).
9. Geick, R., Perry, C. H. & Rupprecht, G. Normal Modes in Hexagonal Boron Nitride. *Phys. Rev.* **146,** 543–547 (1966).
10. Pedersen, T. G., Mera, H. & Nikolić, B. K. Stark effect in low-dimensional hydrogen. *Phys. Rev. A* **93,** 13409 (2016).
11. Ramasubramaniam, A. Large excitonic effects in monolayers of

molybdenum and tungsten dichalcogenides. *Phys. Rev. B* **86,** 115409 (2012).

12. Qiu, D. Y., Da Jornada, F. H. & Louie, S. G. Optical spectrum of MoS_2: Many-body effects and diversity of exciton states. *Phys. Rev. Lett.* **111,** 1–5 (2013).
13. Ugeda, M. M. *et al.* Giant bandgap renormalization and excitonic effects in a monolayer transition metal dichalcogenide semiconductor. *Nat. Mater.* **13,** 1091–1095 (2014).
14. He, K. *et al.* Tightly bound excitons in monolayer WSe_2. *Phys. Rev. Lett.* **113,** 1–5 (2014).
15. Zhang, C., Wang, H., Chan, W., Manolatou, C. & Rana, F. Absorption of light by excitons and trions in monolayers of metal dichalcogenide MoS_2: Experiments and theory. *Phys. Rev. B* **89,** 12–16 (2014).
16. Berkelbach, T. C., Hybertsen, M. S. & Reichman, D. R. Theory of neutral and charged excitons in monolayer transition metal dichalcogenides. *Phys. Rev. B* **88,** 1–6 (2013).
17. Chernikov, A. *et al.* Exciton binding energy and nonhydrogenic Rydberg series in monolayer WS_2. *Phys. Rev. Lett.* **113,** 1–5 (2014).
18. Latini, S., Olsen, T. & Thygesen, K. S. Excitons in van der Waals heterostructures: The important role of dielectric screening. *Phys. Rev. B* **92,** 1–13 (2015).
19. Andersen, K., Latini, S. & Thygesen, K. S. Dielectric Genome of van der Waals Heterostructures. *Nano Lett.* **15,** 4616–4621 (2015).
20. Winther, K. T. & Thygesen, K. S. Quasiparticle band structure engineering in van der Waals heterostructures via dielectric screening. 1–9 (2017).
21. Rasmussen, F. A., Schmidt, P. S., Winther, K. T. & Thygesen, K. S. Efficient many-body calculations for two-dimensional materials using exact limits for the screened potential: Band gaps of MoS_2, *h*-BN, and phosphorene. *Phys. Rev. B* **94,** 155406 (2016).
22. Aivazian, G. *et al.* Many-body effects in nonlinear optical responses of 2D layered semiconductors. *2D Mater.* **4,** 25024 (2017).
23. Chernikov, A., Ruppert, C., Hill, H. M., Rigosi, A. F. & Heinz, T. F. Population inversion and giant bandgap renormalization in atomically thin WS_2 layers. *Nat. Photonics* **9,** 466–470 (2015).
24. Mouri, S. *et al.* Nonlinear photoluminescence in atomically thin layered WSe_2 arising from diffusion-assisted exciton-exciton annihilation. *Phys. Rev. B* **90,** 155449 (2014).
25. Massicotte, M. *et al.* Picosecond photoresponse in van der Waals heterostructures. *Nat. Nanotechnol.* **11,** 42–46 (2015).
